# Optimizing Digital Image Quality for Improved Skin Cancer Detection

**DOI:** 10.3390/jimaging11040107

**Published:** 2025-03-31

**Authors:** Bogdan Dugonik, Marjan Golob, Marko Marhl, Aleksandra Dugonik

**Affiliations:** 1 Faculty of Electrical Engineering and Computer Science, University of Maribor, Koroška Cesta 46, SI-2000 Maribor, Slovenia; marjan.golob@um.si; 2 Faculty of Medicine, University of Maribor, Taborska ulica 8, SI-2000 Maribor, Slovenia; marko.marhl@um.si; 3 Faculty of Education, University of Maribor, Koroška cesta 160, SI-2000 Maribor, Slovenia; 4 Faculty of Natural Sciences and Mathematics, University of Maribor, Koroška cesta 160, SI-2000 Maribor, Slovenia; 5 Department of Dermatology, University Medical Centre Maribor, Ljubljanska Ulica 5, SI-2000 Maribor, Slovenia; aleksandra.dugonik@ukc-mb.si

**Keywords:** dermoscopy, melanoma, color analysis, color error, spectral power distribution, grey card, digital imaging standards

## Abstract

The rising incidence of skin cancer, particularly melanoma, underscores the need for improved diagnostic tools in dermatology. Accurate imaging plays a crucial role in early detection, yet challenges related to color accuracy, image distortion, and resolution persist, leading to diagnostic errors. This study addresses these issues by evaluating color reproduction accuracy across various imaging devices and lighting conditions. Using a ColorChecker test chart, color deviations were measured through Euclidean distances (ΔE*, ΔC*), and nonlinear color differences (ΔE00, ΔC00), while the color rendering index (CRI) and television lighting consistency index (TLCI) were used to evaluate the influence of light sources on image accuracy. Significant color discrepancies were identified among mobile phones, DSLRs, and mirrorless cameras, with inadequate dermatoscope lighting systems contributing to further inaccuracies. We demonstrate practical applications, including manual camera adjustments, grayscale reference cards, post-processing techniques, and optimized lighting conditions, to improve color accuracy. This study provides applicable solutions for enhancing color accuracy in dermatological imaging, emphasizing the need for standardized calibration techniques and imaging protocols to improve diagnostic reliability, support AI-assisted skin cancer detection, and contribute to high-quality image databases for clinical and automated analysis.

## 1. Introduction

Diagnostic procedures in dermatology rely on visually differentiating skin lesions and monitoring their changes over time. Photographic documentation plays a crucial for dermatological diagnostics, management, research, and education [1,2,3,4]. A significant challenge is distinguishing between harmless pigmented lesions and melanoma, a malignant tumor of melanocytes that can be highly pigmented or amelanotic [5]. Early identification of melanoma is vital for patient survival, but visual assessment is often insufficient. Therefore, tools that assess melanoma’s structural and color characteristics are essential.

Dermoscopy, a noninvasive technique introduced in the 1990s, enhances diagnostic accuracy by using a handheld microscope to visualize subsurface skin structures [6]. Accurate color reproduction and high resolution in dermatological images are critical for identifying skin diseases [7,8,9]. The color and structure seen in dermoscopy are influenced by multiple factors, including skin morphology, pathology, and the optical properties of the skin and underlying structures. Common dermatoscopic structures include globules, streaks, net-like patterns, and fingerprint-like ridges. These patterns arise from variations in pigmentation and the interaction of light with skin structures, such as sweat ducts, sebaceous glands, and collagen fiber [10]. The color visible in dermatoscopy is primarily attributed to the presence of melanin, which can be arranged in distinct patterns such as globules, streaks, or dots. The degree of pigmentation—whether it is superficial or deep within the dermis—determines both the intensity and location of the observed color. Melanin colors vary from black and brown to grey and blue, depending on its depth within skin layers [11]. Even subtle hue differences can significantly impact melanoma diagnosis [12]. Additionally, hemoglobin distribution in lesions influences vascular structures and patterns [13]. For example, erythema (redness) results from increased blood flow, while blue areas may indicate deeper vessels or the presence of certain dermal structures.

These features collectively help differentiate benign from malignant lesions and play a crucial role in clinical decision-making. However, among these diagnostic criteria, melanin color remains particularly significant, as variations in pigmentation and vascularization often provide early indications of malignancy. Given the critical role of color in dermoscopic analysis, this study focuses on the accuracy of color reproduction in dermatological imaging. By evaluating color deviations across different imaging devices and lighting conditions, we aim to enhance the reliability of digital dermoscopy and improve diagnostic precision.

Over the past two decades, dermatology has experienced rapid advancements in imaging technologies for skin cancer detection, with a particular emphasis on the accurate identification of melanoma. Melanoma often presents as a lesion with multiple color variations, making its detection challenging. Dermoscopic documentation of melanocytic lesions enables the comparison of current and previous images, facilitating the identification of subtle changes over time and supporting the early diagnosis of melanomas that may not yet exhibit obvious malignancy features [14]. Digital imaging systems such as MoleMax HD, Heine Cube, Visomed, and Fotofinder are widely used for videodermatoscopy and image storage [15]. However, the widespread adoption of these technologies in general practice has been slow, primarily due to concerns over cost and convenience [16,17].

The mobile revolution, marked by the advent of smartphone-based digital cameras and handheld dermoscopes, is driving significant changes in the field of dermatology [18,19]. Based on observations from experienced dermatologists, we hypothesize that different devices produce varying degrees of color reproduction accuracy. Images captured with digital cameras or smartphones often struggle with accurate color rendering [20], with color accuracy varying significantly depending on the camera type, lighting conditions [21], composition, and internal camera components [22]. Furthermore, image processing algorithms may distort color representation, which can negatively impact melanoma screening and potentially lead to incorrect clinical decisions [23]. While professional cameras allow extensive manual adjustments, smartphones are more limited in this regard. However, specialized applications have been developed to enable manual adjustments on smartphone cameras [19].

Smartphones offer a unique opportunity for widespread, accessible, and cost-effective skin cancer screening, particularly for self-monitoring and teledermatology applications. Given the increasing role of artificial intelligence (AI) in dermatological diagnostics, ensuring that images captured by smartphones meet the necessary quality standards is crucial for automated melanoma detection and clinical assessments [24]. Our study explores methods to optimize smartphone-captured images, making them suitable for AI-based analysis as well as for use by dermatologists. By addressing the limitations in color accuracy and standardizing imaging conditions, we aim to enhance the reliability of smartphone-based dermoscopy, ultimately contributing to improved early detection and diagnosis of skin cancer. The challenge with smartphones and digital cameras is that photos of the same object can appear different due to variations in light temperature, white balance (WB) calibration, and image processing, all of which affect color reproduction. Most devices default to an automatic white balance setting, which may not always ensure accurate colors.

Accurate color reproduction is essential for the diagnosis of skin cancer. Several studies have examined dermatological image capture systems in terms of their diagnostic value. In a systematic review, Quigley et al. [25] summarized the existing technologies and technical standards for dermatological images captured using cameras. Similarly, Celebi et al. [26] published a collection of studies presenting methods and techniques for dermatoscopic imaging. Other valuable recommendations are offered by the national guidelines issued by the American Telemedicine Association [27] and the IMI National Guidelines [28], which outline imaging technologies and techniques, emphasizing the importance of camera calibration using a neutral gray reference. A recently published meta-analysis [29] involving 150 dermatologists identified a knowledge gap regarding the use of new camera technologies and photographic techniques for high-quality image acquisition. Baldano et al. suggested that color deviations in dermatological images may result from improper equipment selection, suboptimal lighting conditions, and incorrect photographic techniques [30]. While standardized criteria for acceptable color deviation in dermatological imaging have not yet been established [31], such standards have already been adopted in dentistry [32,33,34].

In this study, we analyze color differences in photos taken with different cameras and smartphones using standardized quantitative image measurement methods to evaluate image quality parameters. Photos of a Color Checker chart photos captured with various digital cameras were exanimated, and image processing software was used to identify color coordinates in different color spaces. Color differences were calculated based on Euclidean distances in the CIELAB color space [35,36].

Additionally, we assessed color reproduction accuracy under different lighting conditions, by employing the color rendering index (CRI) and television lighting consistency index (TLCI) to evaluate consistency and fidelity [37,38,39]. The results were compared with those from a professional digital dermoscopy imaging system to assess the strengths and weaknesses of each device.

The findings reveal significant color discrepancies between mobile phones, high-end consumer cameras, and a professional dermatoscopy system. These discrepancies were largely attributed to the effects of intensive image processing in cameras and inadequate dermatoscope lighting systems, which further contributed to inaccuracies. Improving color reproduction accuracy in cameras is crucial, and proper camera calibration can help mitigate these inconsistences.

Based on our analyses, we present several immediately applicable solutions. We demonstrate how a camera can calculate color temperature more accurately when part of the scene is covered with a white or neutral gray background. White balance calibration critically changes red, green, and blue sensor signals, directly affecting image color. White balance calibration is a standardized practice in dental photography and television production [3,40]. Researchers have investigated computational approaches [41], camera settings [20], and gray card-based WB calibration to achieve better results [42]. Although the properly adjusted color temperature in a camera does not eliminate color deviations, it significantly reduces them. These deviations can be assessed both subjectively and objectively.

Furthermore, we highlight the significance of manual camera adjustments, the utilization of grayscale cards, post-processing techniques, and optimized lighting conditions as effective strategies for improving color accuracy in dermatological imaging. We propose straightforward techniques for calibrating cameras to match the light source of handheld dermatoscopes. This procedure can be performed manually, but in the future, dedicated applications could be developed to automate the adjustment of cameras to meet specific requirements. Achieving this goal requires consolidating knowledge in the field and establishing standardized protocols for skin imaging, enabling reliable comparisons and unified analysis of dermatological images. Thus, our study represents an important step toward raising awareness of the necessity for updated technical standards in dermatological photography. These improvements will enhance skin cancer diagnostics and support the development of high-quality image databases for computer-assisted analysis.

## 2. Materials and Methods

### 2.1. Materials

The devices included in our study comprised the Canon EOS 5DIII digital SLR camera, the Medicam 1000s video dermoscope camera (FotoFinder Systems GmbH, Bad Birnbach, Germany), and high-resolution compact mirrorless cameras, including Canon EOS R7 (Canon Inc., Tokyo, Japan) and Sony 7III (Sony Group Corporation, Tokyo, Japan). Additionally, we evaluated the iPhone 13 (Apple Inc., Cupertino, CA, USA) and the Samsung Galaxy S24 (Samsung Electronics Co., Ltd., Seoul, Republic of Korea).

The color reference test chart used in this study was a 24-color ColorChecker chart (X-Rite Pantone Inc., Grand Rapids, MI, USA), illuminated by an SL150III Daylight LED Studio Video Light (Godox Inc., Shenzhen, China). For dermoscopy image acquisition, we used the handled dermoscope DermLite DL4 (DermLite, 3Gen, Inc., San Juan Capistrano, CA, USA), which was attached to a camera or smartphone via a magnetic ring adaptor. Lighting profiles were measured using the UPRtek MK350 spectrometer (UPRtek, Miaoli, Taiwan). Image and spectral analyses were conducted using a customized software solution developed with functions from Matlab’s Image Processing Toolbox, Photoshop (Adobe Photoshop version 24.6, Adobe Inc., San Jose, CA, USA, 2024), uSpectrum (UPRtek, Miaoli, Taiwan), as well as Adobe CC Software tools (Adobe Inc., San Jose, CA, USA, 2024, Version 24.6.3).

### 2.2. Quantifying Color Differences and Reproduction Accuracy

**Figure 1:**
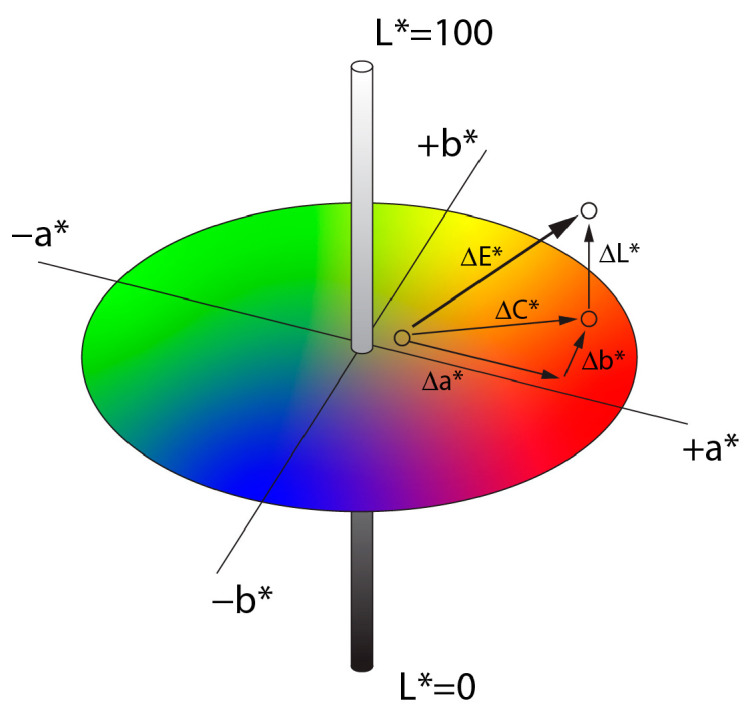
The L*a*b* color space, with a graphical representation of ΔE*, is a device-independent, three-dimensional space designed to be perceptually uniform relative to human vision. The color difference ΔE* measures the Euclidean distance between two colors, while ΔC measures chroma differences.

A significant disparity often exists between the colors represented in a photograph and the actual colors found in nature. This color difference can be quantified to assess the accuracy of color reproduction. The Commission Internationale de l’Eclairage (CIE) has established a classification system based on human visual perception, enabling the measuring of the Euclidean distance within a given color space [36]. The CIELAB or L*a*b* color space provides a device-independent, three-dimensional model for quantifying color discrepancies. As shown in Figure 1, this space is designed to be perceptually uniform, meaning that numerical changes in values correspond to perceived differences in color [43]. The L*a*b* values define: L*—lightness, ranging from 0 (black) to 100 (white), a*—position on the red-green axis, and b*—position on the blue-yellow axis in the chromaticity coordinates [35].

The difference between two colors, known as the color Euclidean distance ΔE*, is calculated as the geometric difference between two color points in the CIELAB color space, using the following formula [44]:

(1) $$∆E*=\sqrt{{\left(∆L*\right)}^{2}+{\left(∆a*\right)}^{2}+{\left(∆b*\right)}^{2}}$$

An individual color is defined by its lightness (L*) and chromatic coordinates (a* and b*). The chroma difference (ΔC*) and the lightness difference (ΔL*) between the two colors are determined by the following equations:

(2) $$C*=\sqrt{{\left(a*\right)}^{2}+{\left(b*\right)}^{2}}$$

(3) $$∆C*=C_{1}^{*}-C_{2}^{*}$$

(4) $$∆L*=L_{1}^{*}-L_{2}^{*}$$

The CIELAB color space, introduced in 1976, is developed to be perceptually uniform, meaning that numerical changes in values should correspond to consistent perceptual differences in color. However, human vision is less sensitive to chroma variations in highly saturated colors than in low-saturation colors, which can cause discrepancies between the computed ΔE* values and the actual perceived color difference [43].

Although two colors may have the same ΔE* value, one may appear more different than the other to the human eye [45]. Hue tolerances are typically more stringent than chroma tolerances, and human vision is more sensitive to changes in specific color ranges. For example, green has a higher tolerance threshold, whereas dark blue exhibits greater perceptual sensitivity. To ensure fair color deviation comparison, the CIE developed more advanced formulas for calculating color differences, with the most recent being CIEDE2000 [46]. The formulas for ΔE00 and ΔC00 are significantly more complex, incorporating perceptual adjustments to better align with human color perception across various industries [46,47]. The CIEDE2000 formula is particularly suited for dermatological imaging, as it improves handling of subtle differences in hue, brightness, and saturation, which are critical for detecting skin conditions. Accurate color representation is essential for identifying inflammation, pigmentation changes, bruising, and lesions, all of which play a key role in diagnosing skin conditions. The CIEDE2000 model incorporates refined algorithms for hue adjustments, which are particularly important for detecting small shifts in skin tone that may indicate pathological changes.

A color deviation of ΔE greater than 1 is perceptible to the human eye [43]. While larger deviations are acceptable in industrial and commercial applications, the standards for medical imaging must be stricter. However, no official standard tolerance has been defined for acceptable color differences in skin imaging [17].

All our results were calculated in the normalized CIELAB color space by standardizing the coordinate values—L* between 0 and 100, a* between −100 and 100, and b* between −100 and 10—resulting in dimensionless quantities for ΔE*, ΔC*, and ΔL*.

### 2.3. Evaluation of Light Source Quality and Color Fidelity

High-quality lighting is crucial for accurate color recognition and reproduction, as well as for visual dermatological examination and capturing high-fidelity images with a camera. Most modern dermoscopy devices utilize white LED light [31]. While LED technology has significantly advanced in recent years, improving color rendering accuracy, inherent differences in spectral composition among various LED light sources are still observed [39].

**Figure 2:**
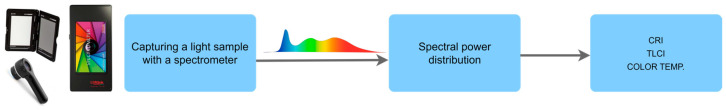
Capturing a light source with a spectrometer. The spectral power distribution graph (SPD), CCT, and qualitative parameters CRI and TLCI were calculated using the uSpectrum software tool.

Figure 2 illustrates the procedure for measuring the various light sources used in our study. Color temperature (CCT), color consistency, and color fidelity were assessed using the color rendering index (CRI) and the television lighting consistency index (TLCI), both derived from the spectral power distribution (SPD) of each light source [37]. CRI measures how accurately a light source reproduces true colors as perceived by the human eye, whereas TLCI assesses how well a light source reproduces colors when captured by a camera [37,39]. The SPD measurements were obtained using a spectrometer, analyzing light reflections from a refe rence gray surface (L = 50, a* = 0, b* = 0).

A handheld dermatoscope offers additional illumination options, including polarized (PD), non-polarized (NPD), and an orange LED light boost. PD light enhances the visualization of deeper skin structures, such as melanin, collagen, and fibrosis, while NPD light is more effective for illuminating superficial structures [31,48]. Functionally, PD and NPD light complement each other in dermatological skin examinations [48]. However, for this study, we limited our measurements to polarized light. Further research is needed to evaluate the impact of different illumination methods on the identification and interpretation of color variations captured using polarized and non-polarized light.

### 2.4. Assessment of Color Deviations in Close-Up and Dermoscopic Imaging

Assessing color deviations in close-up and dermoscopic images is essential for ensuring accurate color reproduction in dermatological diagnostics. Dermoscopy images provide a magnified and detailed view, revealing subsurface skin structure and critical features essential for identifying skin diseases, especially melanoma, whereas close-up images give a general visual representation of skin lesions.

**Figure 3:**
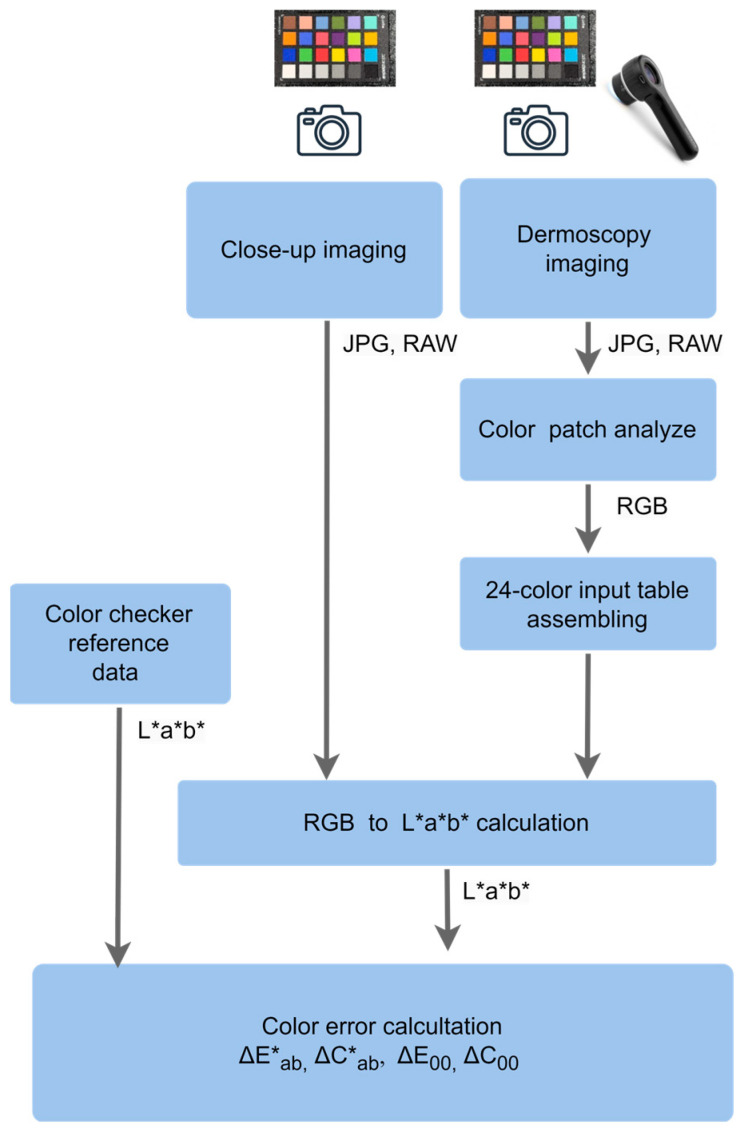
The procedure for assessing color deviations for close-up and dermoscopy images.

The procedural diagram for this assessment is shown in Figure 3, outlining the methodology used to evaluate color differences between these imaging techniques. A 24-color Color Checker chart was placed in a neutral dark gray setting and illuminated with two studio LED lights. For close-up images, the lights were generally set to a color temperature of 5500 K with an intensity of 500 lx. For dermoscopy images, a color temperature of 5800 K was used in close-up mode, while 5000 K was applied in dermoscopy mode. The specific conditions for each measurement are detailed in the Results section. To ensure consistency, the camera parameter settings and color temperature were carefully adjusted. The images were saved in uncompressed (RAW) format and in the highest-quality joint photographic experts group (JPEG) format (lowest compression, typically 95–100% quality or “Fine”/“Super Fine” mode) without additional processing to ensure broad compatibility with clinical and research software while maintaining manageable file sizes. Additionally, PNG format was used exclusively for converting RAW images to facilitate their import into the MATLAB (R2023b) application. This conversion was performed using Adobe Photoshop to maintain high color accuracy during processing.

The cameras were connected to the handheld dermoscope through a magnetic interface to obtain dermoscopy images. Due to the dermoscope’s restricted field of vision (20 mm), color patches from the ColorChecker 24-color chart have to be acquired individually. To evaluate the RGB value of photographed color patches, we use the color picker tool in Adobe Photoshop, enabling the creation of a 24-color target within the ColorChecker dimensions. An alternative approach would have been to use the RezChecker Nano test chart, which allows for the acquisition of all colors in a single step for dermoscopic imaging. However, the target was unavailable during the experiment.

For the color analysis of captured images, we implemented a script in MATLAB using functions from the Image Processing Toolbox. The captured image, stored in TIF, JPG, or PNG format, was read into the MATLAB Workspace using the *function I = imread(name.fmt)*, which loads the image from a graphics file into the variable I. Next, we identified the regions for color analysis using the function: *chart = colorChecker(I, ’Sensitivity’, 0.7).* This step allowed us to detect and define the color patches within the image. We then measured the colors using: *colorTable = measureColor(chart)*. The measured RGB values were subsequently converted into the L*a*b* color space, facilitating a more accurate evaluation of color accuracy. Finally, we calculated color deviation metrics, including CIELAB ΔE* and ΔC*, as well as CIEDE2000 ΔE00 and ΔC00, providing an objective assessment of color differences in the captured images [46,47].

## 3. Results

The results for assessing color deviations in close-up and dermoscopic images are presented for all the devices included in this study. For a complete list of devices and their characteristics, see Section 2: Materials and Methods. To ensure clarity and readability, we clearly differentiate between various devices and specify the lighting conditions used, as the characteristics of both the devices and the light sources significantly impact the resulting image colors. First, we assess color deviations in close-up and dermoscopic images using the Fotofinder Medicam 1000s video camera, a professional dermatology clinic device. For consistency and a systematic approach, we use the same light source as in clinical practice. Second, in the next subsection, we evaluate color deviations in close-up imaging for various devices under studio lighting conditions, specifically a light source with characteristics close to daylight (5500 K, 500 lx). Third, we analyze the results in the context of spectral light characteristics to better understand their influence on color accuracy. Finally, in the last subsection, we present the application of these findings to real melanoma diagnosis and the evaluation of images in a clinical setting.

### 3.1. Color Deviations in Close-Up and Dermoscopic Images Using a Professional Dermatology Device

To establish a reference for color accuracy in dermatological imaging, we first analyze color deviations using the Fotofinder Medicam 1000s, a widely used professional dermoscopy device. This assessment allows us to determine baseline performance under clinical lighting conditions. The dermoscopy device can be used in two different modes: as a close-up imaging tool or in full dermoscopic mode at its highest magnification settings. The latter requires light to pass through additional lenses, which may further affect color accuracy. We assess color deviations in both close-up and fully magnified dermoscopic imaging. In both cases, we use the built-in light source, which has a color temperature of 5000 K in close-up mode and 5800 K in dermoscopy mode.

**Figure 4:**
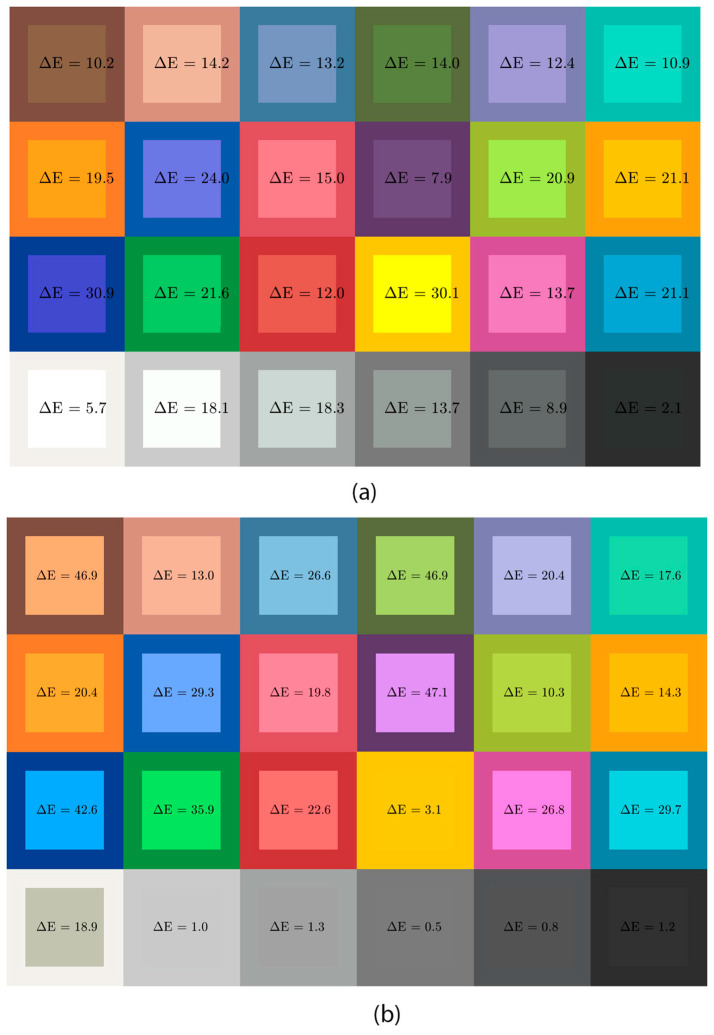
Color variations of 24 reference colors captured with the Medicam 1000s camera in (**a**) close-up and (**b**) dermoscopic imaging modes. The outer squares represent the original reference colors, while the inner squares show the corresponding colors recorded by the camera from the ColorChecker test chart.

The comparison of the obtained results, measured on the reference colors of the ColorChecker from different devices for close-up and dermoscopy images, reveals significant color deviations from the given reference values across all devices in the test. The measured LED illumination characteristics of dermatoscopes contribute substantially to color deviations in dermoscopic images. Figure 4 illustrates the observed color differences in patches from a reference test target, captured in both close-up and dermoscopy images, using the Fotofinder Medicam 1000s video camera. The target was illuminated with the camera’s integrated LED light. The color deviations were visually assessed, ranging from barely noticeable to significantly perceptible. In the close-up image (Figure 4a), color deviations (ΔE) are generally less pronounced but remain visible in grayscale tones, possibly due to less precise camera settings leading to suboptimal illumination. The most significant differences were observed in blue and yellow tones, highlighting the challenges of accurately capturing highly saturated and reflective colors. In contrast, the dermoscopy images (Figure 4b) exhibit more substantial color deviations (ΔE) compared to close-up images. The most significant differences were found in blue, purple, brown, and dark green samples. Neutral tones, light brown, all grays, and black also show notable deviations, suggesting that the camera calibration was effective for brightness and low-saturation samples but less so for highly pigmented colors.

The visual findings in Figure 4 are further quantified by the calculations of ΔE*, ΔC*, ΔE00, and ΔC00, as detailed in the Supplementary Materials (Table S1). In Table 1, we summarize these results by presenting only the average, minimum, and maximum values of ΔE*, ΔC*, and ΔE00. The results indicate that the average color deviations ΔE* are more significant in dermoscopy images (20.7) compared to the close-up images (15.8), demonstrating less accurate color reproduction when using the lens attachment for dermoscopy imaging. The highest color deviation ΔE was recorded in the dermoscopy image for color sample no. 10 (purple, 47.1), whereas in the close-up image, the most significant deviation was observed for the blue color in sample no. 13 (30.9). Significant color differences were also observed for several other color samples in both imaging modes. The lowest deviation was measured in the dermoscopy image for the Neutral5 sample (0.5), with only minor differences noted for other grayscale tones. However, in dermoscopy images, substantial discrepancies were noted for dark skin (46.9), foliage (46.9), blue (42.6), and purple (47.1), according to the color test samples. In the close-up captured images, the most pronounced deviations occurred in yellow (30.1), blue (30.9), and purple-blue (24.0). The substantial differences observed in dark color tones in the dermoscopy images suggest that inconsistent lighting is likely the primary cause of suboptimal outcomes.

Certain colors frequently observed in dermoscopic images, such as sample no. 1 dark skin (ΔE00 = 37.1), sample no. 13 blue (ΔE00 = 39.1), and sample no. 10 purple (ΔE00 = 40.1), exhibited substantial color deviations. Since highly saturated color shades are uncommon in real-life dermatological imaging, we suggest that certain test samples be omitted from the color set, potentially leading to a significant improvement in the results.

The highest chroma deviation (ΔC00) was recorded for color sample no. 1 dark skin (ΔC00 = 11.4). The high ΔE00 values may be attributed to improper lightness parameter settings on the camera, while variations in ΔC00 could result from inadequate calibration of the camera to the LED light’s color temperature and the quality of the LED lighting itself. The measured values indicate significant deviations in color accuracy, while the camera reproduces neutral tones relatively correctly. Precise grayscale tracking suggests accurate calibration of the camera to the color temperature of the light source, which also allows for color correction using a correction matrix. Based on the measurement results, one might conclude that the camera is not diagnostically suitable for dermoscopic imaging. However, since this is a verified and calibrated medical camera, it can be assumed that dermatoscope manufacturers intentionally adjust color rendering to enhance the visual separation of hues. The diagnosis of melanocytic lesions (melanoma) relies on differences in color shades, with white, light brown, dark brown, blue-gray, and black tones being crucial. However, these specific colors exhibit the most significant deviations.

### 3.2. Color Deviations in Close-Up Imaging Across All Devices

To ensure comparability across different imaging devices, we evaluate color deviations in close-up imaging for all devices included in this study (for a complete list of devices and their characteristics, see Section 2: Materials and Methods). The color deviation metrics ΔE*, ΔC*, ΔE00, and ΔC00 were analyzed in images taken under standard studio lighting at 5500 K and dermoscopy illumination for all devices. The results are presented in Table 2. We report color error, chroma differences for the minimum and maximum color deviations, and average values for each device. All cameras exhibit greater color deviations for images taken under dermoscopy illumination. The iPhone 13 shows the highest averages ΔE* (26.5) and maximum deviations ΔE* (55.1), indicating significant challenges in reproducing accurate colors. Both Canon cameras perform better in close-up images, with smaller overall color differences, though they still produce noticeable errors in dermoscopy images. Smartphones show higher color deviations than DSLRs or mirrorless cameras, but they maintain a degree of constancy. Compared to Canon camera models (ΔE* = 16.2 and 19.2), the iPhone 13 exhibited a slightly higher average color deviation (ΔE* = 26.5) under dermoscopic illumination. However, the maximum ΔE* value (55.1) suggests considerable color variation. Significant variances were observed in the Sony camera model, which exhibit high maximum deviation and elevated average values under dermoscopic illumination. This discrepancy is likely due to image processing procedures that do not align well with the dermoscope’s lighting conditions.

For other parameters listed in Table 2, larger deviations in ΔE00 were found for the color samples no. 4 (ΔE00 = 26.4), no. 21 neutral 6.5 (ΔE00 = 23.6), and no. 1 dark skin (ΔE00 = 17.2). The neutral gray samples had a lower average chromatic aberration deviation (ΔC00 = 3.08), suggesting that high ΔE00 deviations result from low illumination quality. The highest ΔC00 values were found in darker color samples, including no. 1 dark skin (ΔC00 = 11.4), no. 3 blue sky (ΔC00 = 13.8), no. 10 purple (ΔC00 = 14.1), and no. 17 magenta (ΔC00 = 18.3).

The comparative analysis of ΔE00 across camera models reveals clear differences in performance. The Canon EOS R7 and 5DIII produced the lowest ΔE00 values, indicating superior color accuracy and stability under both studio and dermoscopic lighting. In contrast, the iPhone 13 and Galaxy S24 recorded the highest deviations, reflecting less consistent color reproduction. The Sony A7III showed moderate deviations, with certain tones demonstrating more substantial shifts, pointing to variability in its internal color processing.

Across all devices, ΔE00 values were consistently higher under dermoscopic LED lighting than under studio lighting, confirming that dermatoscopic conditions introduce greater chromatic inconsistencies. One likely reason is the polarization effect of dermoscopic light, which alters light reflection and absorption at the skin surface. This influences how camera sensors interpret color, particularly in devices with less advanced calibration or color processing—such as smartphones.

Canon cameras demonstrated the most stable performance, making them more reliable for dermatological imaging where precision is critical. In contrast, the professional Medicam 1000s dermatoscope displayed the largest color deviations, likely due to intentional internal processing designed to enhance visual contrast, which can aid diagnosis but reduce color fidelity. Smartphones exhibited broader and less predictable deviations, limiting their clinical accuracy.

Overall, these results highlight the difficulty of maintaining consistent and accurate color reproduction across imaging systems, especially in medical contexts. While chromatic deviations (ΔC00) were generally smaller than total color deviations (ΔE00), this suggests that the tested devices struggled more with achieving correct color balance than with reproducing saturation levels. These findings reinforce the need for improved calibration and lighting optimization to enhance color accuracy in dermoscopic imaging. Although the deviations remain relatively moderate, the increase under dermoscopic conditions suggests that current dermatoscopic LED lights—and additional optics like magnifying lenses—may contribute to the loss of color fidelity.

A more detailed statistical analysis is provided in the Supplementary Materials. Boxplots of ΔE00 color deviations for each camera under dermoscopic (Figure S1) and studio lighting (Figure S2) further support the findings. Notably, (Figure S1) shows that the Medicam 1000s had the highest mean, median, and range of ΔE00 values, statistically confirming that its internal image processing significantly alters color output—potentially enhancing diagnostic visibility at the cost of natural color reproduction.

### 3.3. Influence of Spectral Light Characteristics on Color Accuracy

The color deviation measurements in Table 2 indicate that close-up images captured with all devices exhibit greater color variations under dermoscopy illumination compared to images illuminated with studio lights. Since lighting conditions significantly affect color reproduction, we analyze how different spectral light characteristics influence imaging results. Understanding these variations is essential for achieving consistent and reliable dermatological image analysis.

**Figure 5:**
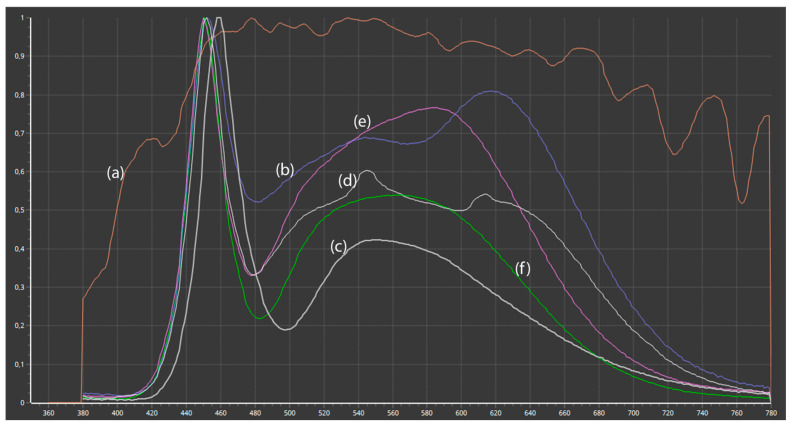
Spectral power distribution graphs of the following light sources: (**a**) Daylight; (**b**) iPhone LED light; (**c**) DermLight DL4 LED light; (**d**) SL150III Daylight studio video LED light; (**e**) Medicam 1000s in a close-up setup; and (**f**) Medicam 1000s used as a dermoscopy LED light.

Figure 5 compares the spectral power distributions (SPDs) of the various light sources used in this study. Despite all employing LED technology, the spectra of artificial light sources exhibit notable differences. All SPDs are expressed in relative units, normalized to 1.0 at 555 nm. Figure 5a illustrates the SPD of daylight, measured at noon, featuring a broad spectrum with uniform intensity across all visible wavelengths. This serves as a baseline for accurately representing natural lighting conditions. Figure 5b presents the SPD of the iPhone 13 white LED, which has a narrower spectrum and a notably high peak in the blue region. Figure 5c shows the spectrum of the DermLite DL4 dermoscopy LED, which has a correlated color temperature (CCT) of 7080 K. It reveals significant peaks at blue (460 nm) and green (550 nm) wavelengths, with reduced intensity in the red spectrum, suggesting potential difficulties in achieving precise color reproduction. Figure 5d illustrates the SL150III Daylight Studio LED, with a CCT of 5500 K, producing a broader, more uniform spectrum that closely resembles daylight. The balanced green and red spectra yield a consistent spectral distribution, making it suitable for professional applications such as television studios [38]. Figure 5e,f present the illumination characteristics of the integrated LEDs in the Medicam 1000s camera. Figure 5e depicts the light used for close-up imaging, and Figure 5f presents the light utilized in dermoscopic imaging.

**Figure 6:**
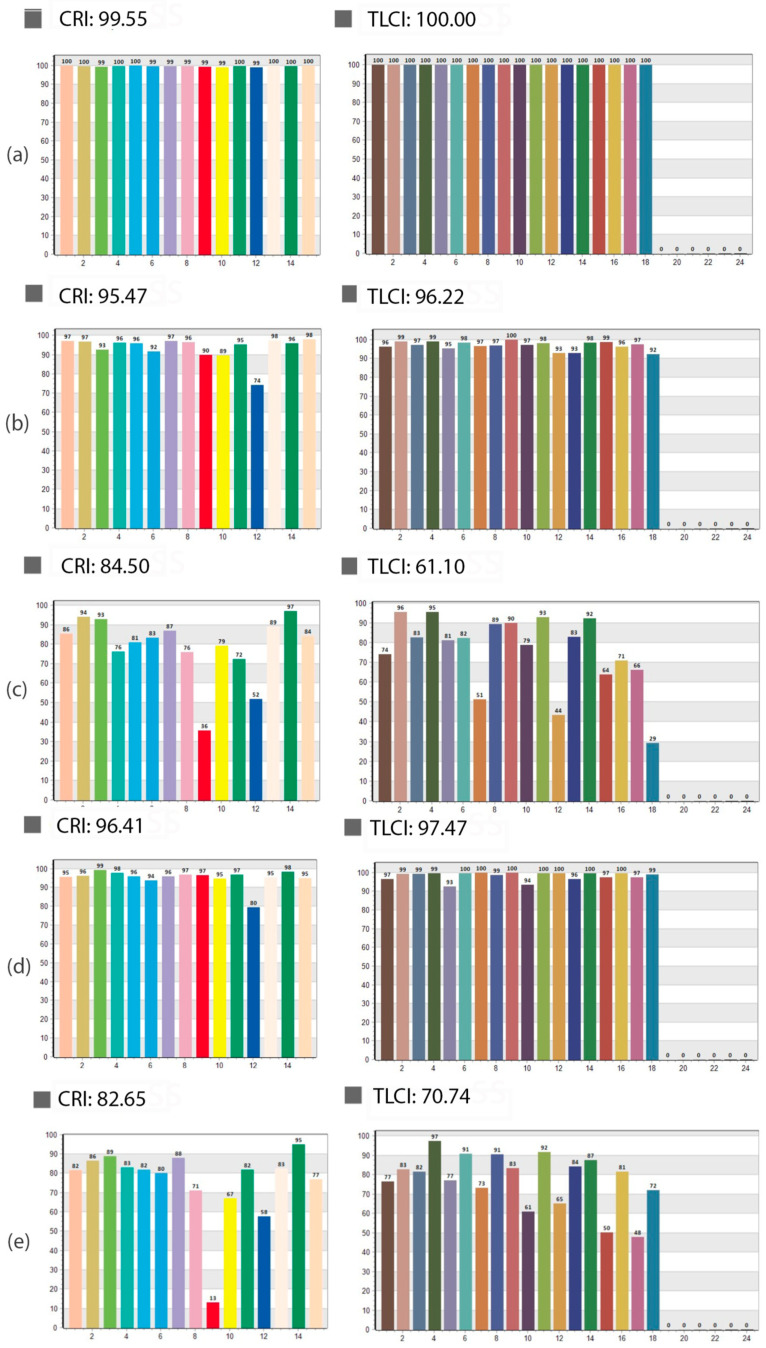
Graphical representation of CRI and TLCI values for the following light sources: (**a**) Daylight; (**b**) iPhone LED; (**c**) Dermlite DL4; (**d**) Godox Studio LED; and (**e**) Medicam 1000s LED. A CRI value closer to 100 indicates near-perfect color rendering, while higher CRI and TLCI values correspond to minimal color deviations and better color accuracy in captured images.

We further analyze the CRI and TLCI values across different light sources, demonstrating significant differences in performance. The results are presented in Figure 6. Daylight exhibited optimal performance, attaining a CRI of 99.55 and an ideal TLCI of 100.00. The results indicate that daylight provides the highest accuracy and uniformity in color rendering (Figure 6a), making it the most suitable reference light source for visual assessments and imaging applications. The iPhone LED demonstrated strong performance, achieving a CRI of 95.47 and a TLCI of 96.22 (Figure 6b), suggesting effective color rendering and high imaging accuracy, though slightly less precise than daylight. The DermLite DL4 LED exhibited significantly lower performance, with a CRI of 84.50 and a TLCI of 61.10 (Figure 6c). These results highlight limitations in color rendering quality, which may compromise its suitability for applications requiring high color fidelity, such as dermatological imaging. The Godox Studio LED demonstrated balanced performance, with a CRI of 96.41 and a TLCI of 97.47 (Figure 6d), closely aligning with daylight values. This makes it a suitable choice for dermatological imaging settings. The Medicam 1000s LED light exhibited slightly higher ratings than the DermLite DL4 dermatoscopy light source, with a CRI of 82.65 and a TLCI of 70.74 (Figure 6e). LED lights in dermatoscopes generally provide suboptimal lighting quality, leading to increased color distortion in dermatoscopic images. A lower CRI and TLCI score, combined with the effects of the magnifying lens, results in reduced color accuracy. However, our results did not demonstrate an absolute correlation between the ΔE values of individual color samples in the image and those obtained from the CRI and TLCI charts.

### 3.4. Application of Image Evaluation to Melanoma Diagnosis

Various devices were used to capture a skin mole, assessing its visual appearance and color variations for perceptual image evaluation. The analysis aims to determine how color differences in images influence the dermatologist’s diagnostic decision-making process. Color differences are visually apparent in the mole images, whether captured close-up or dermoscopically.

For both close-up and dermoscopic imaging, digital medical dermatoscopes are commonly used. Additionally, commercial cameras and smartphones attached to handheld dermatoscopes are increasingly utilized for medical image acquisition. Images can be taken in either automatic or manual mode. While manual mode has the potential to deliver results comparable to those of professional dermatoscopic systems, the camera settings must be properly configured. Therefore, this section demonstrates various techniques for capturing images in manual mode to produce clinically useful results. Only such optimized images can provide clinicians with the level of accuracy required for reliable skin cancer diagnosis.

**Figure 7:**
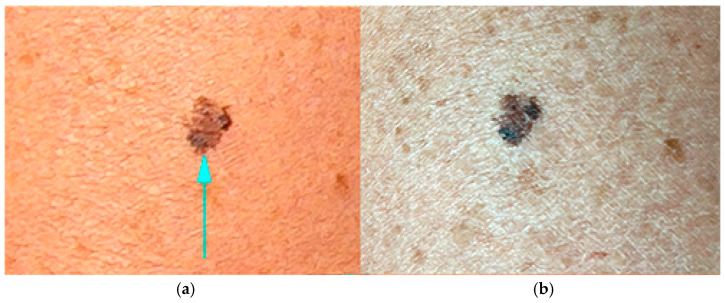
A close-up image of a skin mole captured with (**a**) a Medicam 1000s camera; and (**b**) an iPhone 13.

Figure 7 illustrates the difference in brown tones, which are significantly more pronounced than those visible to the naked eye. Figure 7a, a close-up image taken with the Medicam 1000s camera, shows lighter and more uniform brownish shades, giving the lesion the appearance of a typical benign melanocytic nevus. In contrast, Figure 7b, captured with an iPhone 13, displays more pronounced coloration, with greater contrast between various shades of brown—known as a multicolor appearance, which is a characteristic feature of melanoma. Sunspots are more prominent around the lesion, indicating chronic photodamage to the skin, a factor associated with an increased melanoma risk. The lesion exhibits clinical atypia due to its asymmetry and multicolored appearance; however, these features alone are not sufficient to confirm a melanoma diagnosis. Notably, the images captured with the two different devices significantly alter the visual perception of the lesion compared to direct observation with the naked eye. Differences are particularly evident in the shades of brown within the lesion and in the skin tone of the surrounding area.

**Figure 8:**
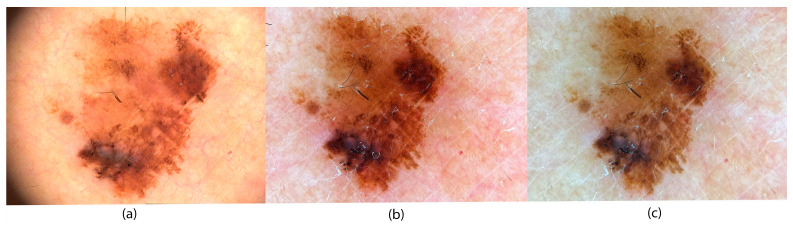
Examples of melanoma exhibiting color variegation: (**a**) Medicam 1000s camera; (**b**) iPhone 13 in manual mode; and (**c**) iPhone 13 in auto mode attached to a DermLite DL4 dermoscope. Accurate color balance is essential for identifying structures specific to melanoma.

In Figure 8, we present a clinical case of melanoma. Figure 8a shows a dermoscopic image captured with the Fotofinder Medicam 1000s camera using default settings, as the system does not allow manual parameter adjustments. Figure 8b,c were captured using an iPhone 13, magnified with a handheld dermatoscope. Figure 8b was taken using manually adjusted camera settings, including ISO sensitivity, shutter speed, and white balance, following calibration with a neutral gray background (CCT = 7080 K, shutter speed of 1/800 s, ISO = 48). Figure 8c shows the same lesion captured in automatic mode with the following settings: ISO = 48, shutter speed 1/400 s, and color temperature 6200 K.

A comparison of Figure 8a–c, performed by a dermatology expert, reveals notable differences in hue and exposure. In Figure 8a, the Medicam 1000s image is characterized by warm, brown-red tones with well-separated brown shades highlighting various dermatoscopic structures. Blood vessels around the lesion are subtly visible in red, giving the surrounding skin a natural, though possibly overexposed, appearance.

In contrast, Figure 8b,c, taken with the iPhone 13, appears darker overall. This difference is clinically relevant: in the well-illuminated upper-left portion of Figure 8a, dermatoscopic features such as globules and streaks are clearly visible, while they are less distinct in Figure 8b,c. Additionally, Figure 8a displays more pronounced color variation (multicolor appearance), making melanoma-specific features more recognizable. The very light brown tones in the upper-left area of Figure 8a, which are absent in Figure 8b,c, could indicate deeper melanin deposits typical of invasive melanoma or be indicative of fair-skinned individuals.

Figure 8b, captured in manual mode, presents the most natural skin tone. Red-colored capillaries are clearly visible around the lesion, forming a mild erythema pattern that is less apparent in Figure 8a, likely due to overexposure. Actinic damage is seen as light brown sunspots, and various brown structures within the lesion are well-defined. However, some finer details, such as small globules in the lower-left area, are less distinguishable than in Figure 8a. The white veil is also less prominent but remains visible.

Figure 8c, taken in auto mode, appears the darkest. The surrounding skin has an unnatural brownish-green hue, and fine telangiectasias are barely visible. While some brown structures can be observed, the overall differentiation is reduced. All brown tones appear darker, which could suggest a superficial melanocytic lesion or melanoma when assessed using dermatoscopic algorithms.

When comparing the three images, Figure 8a,b show greater consistency in brown tones both within and around the lesion. Notably, Figure 8b, captured in manual mode, provides a clinically usable image comparable to the professional Figure 8a. This highlights the importance of using manual settings, as automatic mode (Figure 8c) may compromise critical visual information. Thus, capturing dermatoscopic images in manual mode is essential for accurate and consistent clinical evaluation.

**Figure 9:**
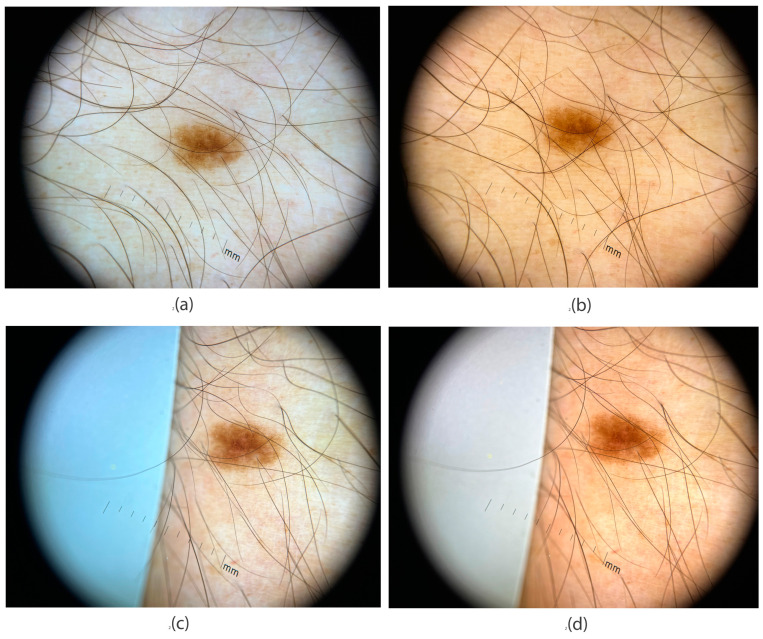
Dermoscopic images captured with an iPhone 13 attached to a DermLite DL4 dermoscope under different camera parameter settings: (**a**) auto mode settings; (**b**) manual mode; (**c**) auto mode with reference grey paper; (**d**) color-corrected image processed in software (Photoshop).

Another example demonstrating how to optimize images for dermatological use is presented in Figure 9. In addition to comparing automatic and manual camera settings, we introduce a more advanced method involving the placement of a reference gray background onto the dermatoscope glass. Image processing software then uses this reference to adjust the illumination and color temperature accurately.

An iPhone 13 attached to a DermLite DL4 dermoscope was used to capture the lesion shown in Figure 9. In Figure 9a, the image was taken in automatic mode, with the camera selecting all parameters: shutter speed 1/400 s, ISO 48, and color temperature 6200 K. The resulting image appears visually overexposed, with a dominant green hue, resembling dermoscopic images captured with non-polarized light.

Figure 9b was captured using manual settings, based on values measured with a light meter and grayscale card (CCT = 7080 K, shutter speed = 1/800 s, ISO = 48). These settings were saved in the Yamera Photo App for future use. The brown tones within the lesion are well-defined, and the surrounding skin appears in a natural pinkish-brown shade.

In Figure 9c, a gray reference sheet (L = 50, a* = 0, b* = 0) was included in the image frame, partially covering the dermatoscope glass. This reference was used in post-production to calibrate the image’s color temperature and brightness. The resulting image shows well-separated brown tones in the lesion, though the surrounding skin appears very light and slightly unnatural.

Figure 9d presents the final color-corrected version processed in Photoshop, using the gray reference data from Figure 9c. The correction parameters can be saved as a user profile for future image processing. In this version, the lesion’s brown tones are clearly distinguishable, and the surrounding skin appears in natural pinkish-brown hues, closely resembling real-life observation.

Taken together, the analysis of Figure 8 and Figure 9 illustrates a clear pathway for obtaining diagnostically useful images in dermatology. The simplest and most practical procedure, easily applicable with a smartphone, is directly derived from the manual settings used in Figure 8b and Figure 9b, where the following values were applied: CCT = 7080 K, shutter speed = 1/800 s, and ISO = 48.

To make the procedure explicitly clear, we propose the following calibration guideline for smartphone cameras, which involves adjusting three key parameters:

- Set white balance to CCT = 7080 K
- Set ISO to ISO = 48
- Set shutter speed to 1/800

The choice of white balance (CCT = 7080 K) is based on measurements of the dermatoscopic light characteristics (Figure 5c). A low ISO value is recommended to minimize image noise and ensure sharpness, which is essential for accurate dermatoscopic evaluation. The shutter speed of 1/800 s was determined by measuring exposure on a mid-gray color patch (L = 50, a* = 0, b* = 0). This value ensures proper exposure, especially considering that smartphone cameras have fixed apertures. If the intensity of the lighting conditions differs, we recommend adjusting the shutter speed accordingly. This requires only minimal practice and is relatively simple to implement.

A more advanced method, illustrated in Figure 9c,d, involves placing a reference gray background onto the dermatoscope glass. This approach requires more technical expertise and is not currently recommended for everyday clinical use. However, it is shown here for its research relevance. In the future, this method could be integrated into user-friendly software that automates color correction and exposure adjustments. Such software would detect the reference gray value and automatically calibrate illumination and color temperature during post-processing, providing higher precision and clinical utility.

This process—for capturing close-up and dermoscopic images with a DSLR, mirrorless camera, or smartphone—could be incorporated into a fully automated system, as schematically illustrated in the Supplementary Materials (Figure S3). Automating this workflow would enhance diagnostic accuracy and significantly improve image comparability. This advancement would also facilitate the development of AI-based tools, offering considerable support for dermatologists in clinical practice.

## 4. Discussion

Our study contributes to the improved use of dermoscopy and skin imaging, which has become an essential tool in daily clinical practice. Enhancing techniques for capturing high-quality dermoscopic images is crucial to ensure accurate representation of skin lesion colors and structures. Color deviations, particularly in brown melanin deposits, must be minimized, as they may lead to overdiagnosis (e.g., a benign lesion mistakenly classified as malignant) or misdiagnosis (e.g., a malignant lesion assessed as benign or superficial). Furthermore, accurate color reproduction is essential for monitoring lesion evolution over time. Our results indicate that color deviations in dermoscopic images vary across devices, with smartphone cameras exhibiting the highest deviations, while professional cameras achieved slightly better results. However, even the professional dermatoscope camera (FotoFinder) demonstrated noticeable color inaccuracies.

The presented approach to enhancing dermoscopic imaging focuses on reducing color discrepancies through two proposed methods. The goal of these methods is to strike a balance between minimal time investment for implementation, ease of use, and maximum improvement in color accuracy. The first method demonstrated that manually setting camera parameters to known light source values significantly reduced color differences over time. The second method involved placing a gray card in the scene, allowing for software-based color matching and improved consistency across images.

Color reproduction is highly dependent on the light source’s color temperature, which can be neutralized by WB adjustments. Several studies [21,31,37,38,39] have highlighted the impact of illumination quality on color response accuracy. Color consistency is also critical in the AI-based deep learning algorithms increasingly used for dermatological diagnoses [22,49,50,51,52]. Since AI models rely on color information extracted from dermoscopic images, standardized lighting conditions are essential for ensuring consistent input data [52]. Our SPD, CRI, and TLCI analyses revealed limitations in the white LED light sources used in dermatoscopes. The handheld dermatoscope’s LEDs demonstrated higher color deviations due to uneven SPD and low CRI/TLCI values. To enhance color accuracy, future dermoscope designs should incorporate optimized LED lighting, ensuring standardized spectral characteristics and transparent disclosure of SPD, CCT, and TLCI values [31].

Proper camera settings further improve image color accuracy. While professional cameras allow precise parameter adjustments, they are often time-consuming to configure. Conversely, smartphone cameras, with smaller sensors, attempt to enhance image quality through automatic image processing [52]. However, intensive image processing can result in incorrect color representation, significantly impacting clinical decision-making. Nevertheless, dedicated smartphone applications allow for manual camera settings, enabling image quality that rivals high-end cameras. Additionally, smartphones provide integrated tools for image processing, transfer, and documentation, alongside AI-powered applications that simplify image capture and analysis. Our findings suggest that smartphones already possess strong potential to replace high-quality consumer cameras for dermoscopic imaging, if color calibration techniques are implemented effectively. To further reduce color differences between images captured with professional dermatoscopes and mobile phones, it would be necessary to develop dedicated image acquisition applications that would directly adjust colors using LUT tables for smartphone images. For this purpose, L*a*b* color values would be required for a standardized set of colors, which are commonly used for color correction in professional digital dermatoscopes.

Standardized protocols for color accuracy in medical imaging are crucial for regulatory validation and technological advancement [30,53,54]. The sheer volume of images captured in dermatology does not guarantee high image quality, nor does it adhere to the Digital Imaging and Communications in Medicine (DICOM) standard [54]. While DICOM is widely used in radiology, cardiology, and radiotherapy, its adoption in dermatology remains limited. However, growing recognition of DICOM’s importance in dermatologic imaging is prompting efforts to improve image quality (spatial resolution, color accuracy, sharpness), patient data security, and interoperability [25]. Based on our findings, we propose that DICOM standards for dermatology should include camera setting descriptions for recording dermatoscopic imaging, along with essential metadata on light source characteristics and color temperature.

Furthermore, our study presents practical solutions for immediate implementation. We demonstrate that a camera can more accurately calculate color temperature when a portion of the scene includes a white or neutral gray reference background. White balance calibration plays a critical role in adjusting red, green, and blue sensor signals, thereby enhancing image color fidelity. Similar practices are already standardized in dental photography and television production [3,40]. Research has explored computational approaches [41], optimized camera settings [20], and gray card-based WB calibration to achieve better color accuracy [42]. Although correctly setting the camera’s color temperature does not eliminate all color deviations, it significantly reduces them, making both subjective and objective color assessments more reliable.

Our findings emphasize the importance of manual camera adjustments, grayscale references, post-processing techniques, and optimized lighting conditions as effective measures for improving color accuracy in dermatological imaging. We propose straightforward methods for calibrating cameras to match handheld dermatoscope light sources. While this calibration process can be performed manually, future innovations could enable automated adjustments through dedicated applications. Achieving standardized skin imaging protocols would allow for more reliable comparisons and a unified framework for analyzing dermatological images. By raising awareness of the need for technical standards in dermatological photography, this study contributes to improving skin cancer diagnostics and supporting the development of high-quality image databases for AI-assisted analysis.

## 5. Conclusions

Our study presents novel solutions for improving image quality, standardization, and automation in dermatological photography, with a particular emphasis on laying the groundwork for future AI applications in skin cancer diagnostics. Color accuracy in dermoscopic imaging is essential for clinical decision-making, lesion monitoring, and the development of reliable AI algorithms for automated melanoma detection. However, our findings show that significant color discrepancies persist due to device limitations, inconsistent illumination, and the use of automatic camera settings—all of which can compromise diagnostic precision. Through systematic analyses of color reproduction, we highlight the need for standardized, high-quality LED light sources to ensure consistent imaging conditions. Our results demonstrate that automatic smartphone settings are not ideal for producing clinically useful dermatological images. In contrast, manually adjusting camera parameters—specifically, based on known lighting conditions—significantly reduces color deviations and yields images more suitable for clinical diagnosis. A more advanced technique, involving the use of a grayscale reference card in the image frame, allows for even greater color accuracy and improved clinical outcomes. However, this method requires additional technical knowledge and is not currently feasible for routine use in clinical practice. This underscores the need for further research and development in this area. Looking forward, such advanced calibration methods could be integrated into user-friendly smartphone applications that automate color correction, exposure adjustment, and other essential settings. These tools would not only improve diagnostic accuracy but also support the development of AI-assisted dermatological diagnostics. Beyond individual image optimization, we stress the urgent need for standardization in dermatological photography. Establishing unified technical requirements—including camera settings, lighting specifications, and file storage protocols—within dedicated imaging software would ensure consistency across clinical environments. This, in turn, would enable reliable image comparison, facilitate automated analysis, and support the creation of high-quality image databases for AI training. By implementing standardized imaging protocols and automation techniques, we can significantly enhance the reproducibility and reliability of digital dermoscopy, ultimately advancing both clinical diagnostics and AI-driven dermatological analysis.

## Figures and Tables

**Table 1:** Evaluation and comparison of close-up and dermoscopy images using the Fotofinder Medicam 1000s. The average, minimum, and maximum values are presented for ΔE*, ΔC*, ΔE00, and ΔC00.

	Close-Up Images				Dermoscopy Images			
	ΔE*	ΔC*	ΔE00	ΔC00	ΔE*	ΔC*	ΔE00	ΔC00
Avg	15.8	9.2	10.9	4.6	20.7	9.6	16.2	4.4
Min	2.1	1.1	3	0.8	0.5	0.2	0.5	0.2
Max	30.9	29.1	16	10.2	47.1	29.1	40.1	11.4

**Table 2:** Evaluation of camera models in accurately capturing colors under various lighting conditions. The evaluation metrics comprise total color deviation as per the CIELAB model (ΔE*), chromaticity deviation in the CIELAB model (ΔC*), the contemporary and more perceptually precise color deviation metric from CIEDE2000 (ΔE00), and chromaticity deviation (ΔC00). The average deviation values for the specified metrics across all 24 color samples are presented.

Camera Model		Studio Light CCT 5500 K				Dermoscope LED Light			
		ΔE*	ΔC*	ΔE00	ΔC00	ΔE*	ΔC*	ΔE00	ΔC00
Canon EOS R7	Aver.	11.5	9.7	6.3	4.7	16.2	12.2	8.7	5.1
	Min	3.6	0.5	2.4	0.5	0.8	0.4	1	0.4
	Max	24.7	24.6	12.9	9.5	34.4	33.3	17.1	14.3
iPhone 13	Aver.	18	11.2	11.8	4.7	26.5	21.8	12.3	7.5
	Min	2.6	0.7	1.9	0.8	3.8	2.2	3.4	1.3
	Max	35.2	34.2	22.7	10.1	55.1	55	26.4	18
Canon EOS 5DIII	Aver.	10.9	8.3	6.2	4	19.6	16.7	9.6	7
	Min	1.1	1.1	0.8	0.8	1.9	1.9	2.2	2.2
	Max	18.8	18.6	9.8	8.7	40.3	39.5	15.2	13.5
Galaxy S24	Aver.	17.4	15.3	9.6	7.5	16.8	9.7	12.1	5.3
	Min	5.7	1.6	4.2	0.7	4.5	0.2	3.1	0.1
	Max	43.6	43.2	19.8	18.4	37.8	32	23.2	19.7
Sony A7III	Aver.	12.3	8.9	7.3	3.7	24	19.6	11.5	7.3
	Min	1.7	0.3	1.2	0.5	3.8	2.2	3.4	1.3
	Max	32	31.2	15.3	9.6	55.7	55	34	32.6

## Data Availability

The original contributions presented in this study are included in the article. Further inquiries can be directed to the corresponding author.

## Supplementary Materials

The following supporting information can be downloaded at https://www.mdpi.com/article/10.3390/jimaging11040107/s1, Table S1: Values of the 24 reference colors of the ColorChecker test target, along with their corresponding L*a*b* values and the calculated color deviations for close-up and dermoscopy images for ΔE*, ΔC*, ΔE00, and ΔC00. Figure S1: Boxplot of ΔE00 color deviations across camera models under dermoscopic lighting conditions; Figure S2: Boxplot of ΔE00 color deviations across camera models under studio lighting conditions. Figure S3: The algorithm for capturing close-up and dermoscopy images with color correction.
