# Supplementary material for: Optimizing Digital Image Quality for Improved Skin Cancer Detection
Source: J Imaging. 2025 Mar 31;11(4):107. doi: 10.3390/jimaging11040107 (PMC12027704; doi:10.3390/jimaging11040107)
Supplement: Supplementary file 1 [file jimaging-11-00107-s001.zip › jimaging-3502446-supplementary.pdf]

Journal of Imaging

MDPI

Section S1: Corresponding measured L*a*b* color values for Medicam 1000s camera

Table S1 Values of the 24 reference colors of the ColorChecker test target, along with their corresponding L*a*b* values and the calculated color deviations for close-up and dermoscopy images for ΔE*, ΔC*, ΔE00, and ΔC00.

Xrite Color Checker Lab data					Close-up image				Dermoscopy image			
**ROI**	**Color**	**L**	**a**	**b**	**ΔE***	**ΔC***	**ΔE00**	**ΔC00**	**ΔE***	**ΔC***	**ΔE00**	**ΔC00**
1	DarkSkin	37.5	14.4	14.9	10.2	7.3	8.8	6.1	46.9	26.4	37.4	11.4
2	LightSkin	64.7	19.3	17.5	14.2	6.6	10.9	5.1	13	4.9	9.9	3.5
3	BlueSky	49.3	-3.8	-22.5	13.2	5.5	12.3	4.8	26.6	4.3	22.6	3.7
4	Foliage	43.5	-12.7	22,7	14	11,6	9,3	5,3	46,9	29,1	3.2	10.3
5	BlueFlower	54.9	9.6	-24.8	12.4	6.3	9.8	3.2	20.4	0.7	16.8	0.3
6	BluishGreen	70.5	-32.3	-0.4	10.9	3.3	7.8	2	17.6	15.1	11	8.9
7	Orange	62.7	35.8	56.5	19.5	15.9	11.4	7.1	20.4	15.7	12.7	7.7
8	Purplish Blue	39.4	10.8	-45.2	24	19.5	15.7	7.8	29.3	2.4	28.1	1
9	ModerateRed	50.6	48.6	16.7	15	4.9	13	1.6	19.8	5.7	17	3
10	Purple	30.1	22.5	-20.9	7.9	1.2	6.4	0.8	47.1	26.2	40.1	8.8
11	YellowGreen	71.8	-24.1	58.2	20.9	14.5	11.8	5.4	10.3	3.7	6.9	1
12	OrangeYellow	71.5	18.2	67.4	21.1	18.5	10.1	7	14.3	11.5	7.4	4.2
13	Blue	28.4	15.4	-49.8	30.9	29.1	12.7	9.6	42.6	16.7	39.9	9.4
14	Green	54.4	-39.7	32.3	21.6	10.9	15.9	3.1	35.9	23.2	22.6	6.4
15	Red	42.4	51.1	28.6	12	5.1	10.9	2	22.6	3.2	21.7	1.3
16	Yellow	81.8	2.7	80.4	30.1	25.8	14	10.2	3.1	3.1	1.2	1.2
17	Magenta	50.6	51.3	-14.1	13.7	4.5	12	2.1	26.8	17.6	18.9	6.9
18	Cyan	49.6	-29.7	-28.3	21.1	15.4	16	8.9	29.7	10.6	24.2	6.6
19	White	95.2	-1.0	2.9	5.7	3.1	4.2	3.1	18.9	8.1	12.9	6.8
20	Neutral8	81.3	-0.6	0.4	18.1	1.1	11.4	1.5	1	0.7	1.1	0.9
21	Neutral6.5	66.9	-0.8	-0.1	18.3	3.9	13.9	4.9	1.3	0.8	1.4	1.1
22	Neutral5	50.8	-0.1	0.1	13.7	3	12.9	4.1	0.5	0.2	0.5	0.2
23	Neutral3.5	35.6	-0.5	-0.5	8.9	1.7	8.1	2.4	0.8	0.7	0.9	0.8
24	Black	20.6	0.1	-0.5	2.1	2.1	3	3	1.2	0.5	0.9	0.5

Section S2: Statistical Analysis of ΔE00 Color Deviations Across Camera Models

A statistical analysis of color deviations was conducted for the professional Fotofinder Medicam 1000s and other camera models under two lighting conditions: dermoscopic illumination (Figure 1S) and studio lighting (Figure 2S).

3.1 Statistical Analysis of Color Deviations Across Camera Models under Dermoscopic Light

The results of the statistical analysis under dermoscopic light are presented in Figure 1S. Among all tested devices, the Fotofinder Medicam 1000s exhibited the highest mean, median, and range of ΔE00 values, indicating the most substantial color deviations. This likely reflects the use of intensive internal color processing algorithms, which may intentionally alter colors to enhance visual contrast, thereby aiding dermatological diagnosis—though at the expense of natural color fidelity. Canon cameras demonstrated the most accurate color reproduction, characterized by low ΔE00 values. The Sony A7RIII showed greater variability and positive skewness, indicating that while it generally performs well, it occasionally produces pronounced color shifts in certain tones. The Samsung S24 exhibited the lowest standard deviation and a narrow deviation range, suggesting consistent and predictable color reproduction, though not necessarily the most accurate. The iPhone 13 displayed moderate performance, with low variability but a higher mean ΔE00 than the Canon cameras. Smartphones typically employ complex, embedded color correction algorithms, making it more difficult to adjust their profiles without accessing deeper system-level settings.

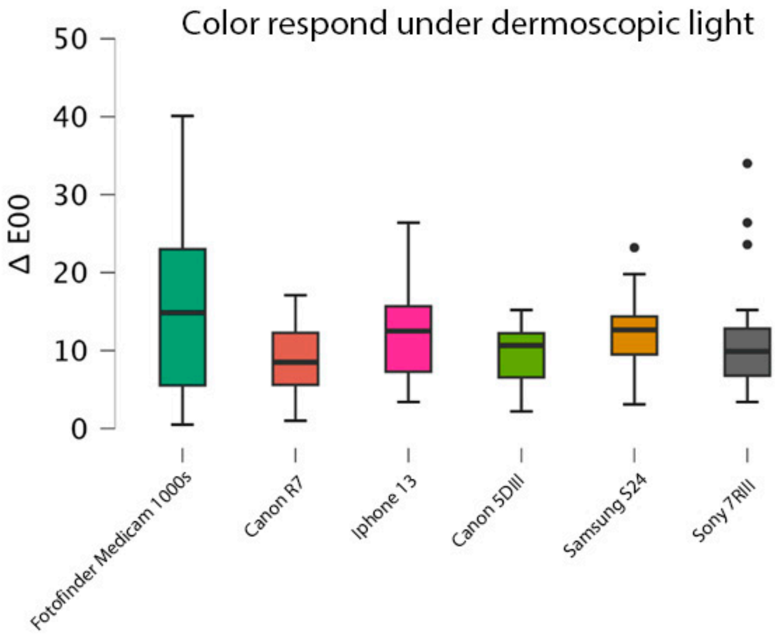

Figure S1 Boxplot of ΔE00 color deviations across camera models under dermoscopic lighting conditions.

Average Color Deviation (Mean): Fotofinder Medicam 1000s (16.22) exhibits the highest average ΔE00, indicating the most significant color deviations and Canon 5DIII (9.56) has the lowest mean value, suggesting more accurate color reproduction.
Median (50th Percentile): The highest median is found in Fotofinder Medicam 1000s (14.85), while the lowest is in Canon 5DIII (10.65).
Standard Deviation (Color Deviation Variability) - Medicam 1000s (12.92) has the highest standard deviation, showing a broad spread of color deviations and Samsung S24 (4.86) has the lowest standard deviation, meaning more stable and predictable color reproduction.
Range (Maximum - Minimum Deviation) - the widest range is observed in Medicam 1000s (39.6), indicating large variability in color deviations, Samsung S24 (13.00) has the smallest range, meaning more consistent results.
Skewness (Asymmetry of Data Distribution) - Canon R7 (-0.068) and Canon 5DIII (-0.279) show slight negative skewness, suggesting more values in the higher ΔE00 range. Sony A7RIII (1.695) has the highest positive skewness, indicating that most color deviations are small, but a few extreme values significantly increase the overall average.
Mode (Most Frequently Occurring Value) - Medicam 1000s (9.900) and Sony A7RIII (9.900) share the same most frequently occurring ΔE00 value; iPhone 13 (3.400) has the lowest mode, meaning a large portion of its color deviations are concentrated around lower values.

3.2 Statistical Analysis of Color Deviations Across Camera Models under Studio Light

The statistical analysis of color deviations for the professional Fotofinder Medicam 1000s and smartphone cameras under studio lighting is presented in Figure 2S. The Fotofinder Medicam 1000s shows notable deviations, likely resulting from intentional internal color processing. Both Canon camera models continue to demonstrate the highest color accuracy, consistent with the results observed under dermoscopic lighting. In contrast, smartphones exhibit more variable performance, with the highest color deviations recorded among the tested devices.

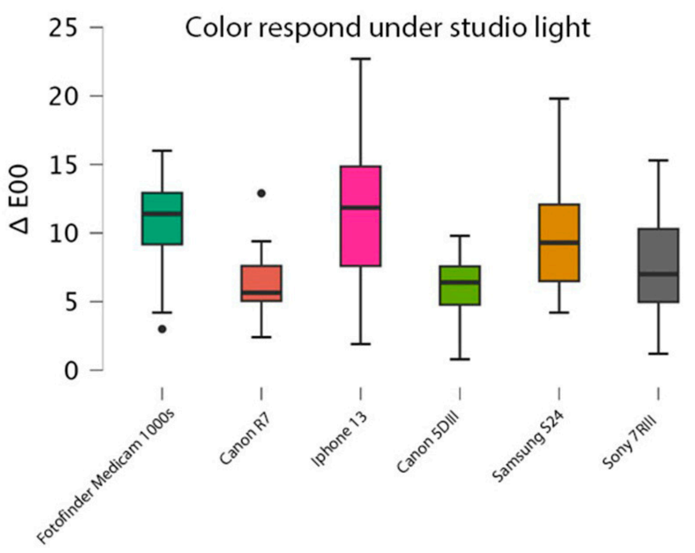

Figure S2 Boxplot of ΔE00 color deviations across camera models under studio lighting conditions.

Average Color Deviation (Mean) - Canon 5DIII shows the lowest mean ΔE00 (6.221), indicating the best color accuracy, iPhone 13 has the highest mean ΔE00 (11.750), suggesting the largest color deviations, and Fotofinder Medicam 1000s also has a high mean ΔE00 (10.929), indicating significant color shifts.

Median - the median follows a similar pattern as the mean, with Canon 5DIII (6.400) having the lowest median and iPhone 13 (11.850) the highest. Canon R7 has a relatively low median (5.650), confirming its stable color accuracy.

Standard Deviation (Color Deviation Variability) - Canon 5DIII (2.242) and Canon R7 (2.273) have the lowest standard deviations, meaning they are the most consistent in color reproduction, Samsung S24 (3.818) and iPhone 13 (5.201) have the highest standard deviations, indicating larger color deviations and less consistency.

Range (Maximum - Minimum Deviation) - Canon 5DIII (Min: 0.800, Max: 9.800) has the smallest range, showing very stable color performance, iPhone 13 (Min: 1.900, Max: 22.700) has the largest range, indicating significant variability. Fotofinder Medicam 1000s (Min: 3.000, Max: 16.000) also shows a wide range, it applies heavy color processing.

Skewness (Distribution Shape)

Most cameras have near-zero skewness, meaning balanced distributions of color deviations. Fotofinder Medicam 1000s (-0.641) is negatively skewed, suggesting a higher concentration of lower ΔE00 values but with some extreme higher values**.**

Section S3: Algorithm for automating the capture of close-Up and dermoscopy images with color correction

The process for capturing close-up and dermoscopy images using a DSLR, mirrorless camera, or smartphone can be automated as schematically presented in Fig. 1S. The color temperature can be assessed in postproduction by analyzing the reference grayscale card under chosen lighting conditions. Images can be stored in an uncompressed format, such as RAW files, or a compressed format, such as JPG or HEIF (high efficiency image format). HEIF is not as widely supported by computer software and online platforms, and it therefore often requires conversion to a JPG. As a result, JPG remains the most universally accepted file format [18]. Uncompressed images are expected to exhibit lower color variation but require significantly more storage capacity. Although several studies have confirmed that manual adjustment of camera parameters for exposure and color temperature improves image quality and reduces color variation, in dermatology clinics, images are still mostly captured in automatic mode and stored in compressed JPG format.

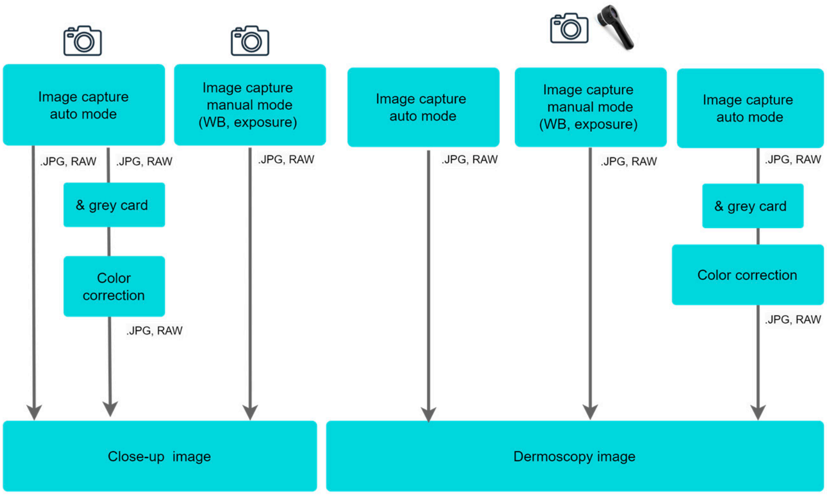

Figure S3 The algorithm for capturing close-up and dermoscopy images with color correction.
